# Weakest-Link Dynamics Predict Apparent Antibiotic Interactions in a Model Cross-Feeding Community

**DOI:** 10.1128/AAC.00465-20

**Published:** 2020-10-20

**Authors:** Elizabeth M. Adamowicz, William R. Harcombe

**Affiliations:** a Department of Genetics, Cell Biology, and Development, University of Minnesota, Minneapolis, Minnesota, USA; b Department of Ecology, Evolution and Behavior, University of Minnesota, St. Paul, Minnesota, USA; c BioTechnology Institute, University of Minnesota, St. Paul, Minnesota, USA

**Keywords:** antibiotic resistance, drug interactions, microbial communities

## Abstract

With the growing global threat of antimicrobial resistance, novel strategies are required for combatting resistant pathogens. Combination therapy, in which multiple drugs are used to treat an infection, has proven highly successful in the treatment of cancer and HIV. However, this practice has proven challenging for the treatment of bacterial infections due to difficulties in selecting the correct combinations and dosages. An additional challenge in infection treatment is the polymicrobial nature of many infections, which may respond to antibiotics differently than a monoculture pathogen. This study tests whether patterns of antibiotic interactions (synergy, antagonism, or independence/additivity) in monoculture can be used to predict antibiotic interactions in an obligate cross-feeding coculture. Using our previously described weakest-link hypothesis, we hypothesized antibiotic interactions in coculture based on the interactions we observed in monoculture. We then compared our predictions to observed antibiotic interactions in coculture. We tested the interactions between 10 previously identified antibiotic combinations using checkerboard assays. Although our antibiotic combinations interacted differently than predicted in our monocultures, our monoculture results were generally sufficient to predict coculture patterns based solely on the weakest-link hypothesis. These results suggest that combination therapy for cross-feeding multispecies infections may be successfully designed based on antibiotic interaction patterns for their component species.

## Abstract (precis)

With the growing global threat of antimicrobial resistance, novel strategies are required for combatting resistant pathogens. Combination therapy, in which multiple drugs are used to treat an infection, has proven highly successful in the treatment of cancer and HIV. However, this practice has proven challenging for the treatment of bacterial infections due to difficulties in selecting the correct combinations and dosages. An additional challenge in infection treatment is the polymicrobial nature of many infections, which may respond to antibiotics differently than a monoculture pathogen.

## INTRODUCTION

Antibiotic resistance is a growing global threat. In the United States alone, an estimated 2.8 million antibiotic-resistant infections occur every year (1). Many previously treatable infections, such as tuberculosis (2), urinary tract infections (3), and even *Staphylococcus*-mediated skin infections (4) now require higher doses of more powerful antibiotics, and the development of novel antimicrobials is limited (5, 6). One potential alternative treatment strategy is the use of drug combinations, which have been used successfully in HIV treatment (7, 8) and cancer chemotherapy (9, 10). In cases of bacterial infections, multidrug therapy has been adopted in only a few specific infections, such as treatment for drug-sensitive tuberculosis (2) and use of trimethoprim-sulfamethoxazole to treat skin and soft tissue infections (11). However, clinical trials of combination therapy in the treatment of bacterial infections in patients have been limited. Choosing the correct drug combination is difficult (12, 13), and efficacy has been mixed (14, 15). A greater understanding of the mechanisms driving effective combination therapy are therefore required for successful clinical implementation.

The success of combination therapy is affected by interactions between drugs, in which the activity and effectiveness of one drug is impacted by the presence or absence of another (16) There are several mechanisms by which antibiotics may synergize (work more effectively or at lower doses together than separately) or antagonize (work less effectively or at higher doses together than separately). While the precise nature of these interactions depends on the drugs and the bacterial species being targeted, some general mechanisms have been described for different classes of antibiotics (17). Synergistic interactions tend to occur when one drug facilitates cellular entry (18–20) or increased efficacy (21) of another, or when the drugs target similar cellular processes (22, 23). Conversely, antagonism may occur when one antibiotic induces tolerance or resistance to another (17, 24, 25), or when one drug corrects for the physiological disruptions caused by another (26). These are general trends only, however, and many species- and drug-specific exceptions apply, making it challenging to predict drug interactions *a priori* in new systems.

It is increasingly appreciated that bacterial infections are often polymicrobial. Numerous clinically relevant infections are now known to involve multiple species, consisting of a single pathogen and various commensal partners, or several coinfecting pathogens (27, 28). Polymicrobial infections have been observed to have worse clinical outcomes in some cases (29–31), although these results are mixed (32, 33). The metabolic interactions (both positive and negative) among these species have been demonstrated to impact antibiotic response (34). One such positive interaction is cross-feeding, in which one species produces an essential metabolite for another; this also occurs in infection contexts (35). For example, in a cystic fibrosis model where the pathogen Pseudomonas aeruginosa depends on the mucin degradation products supplied by a community of anaerobic commensals, antibiotics specifically targeting the anaerobes decreased P. aeruginosa abundance despite its intrinsic resistance to the antibiotic (36). Treatment regimens might, therefore, be more effective if metabolic interactions among species are taken into account; however, little research has been done on how cross-feeding might impact combination therapy.

To this end, we aimed to test whether cross-feeding interactions in a model bacterial community influence antibiotic interactions. We selected 10 combinations of six antibiotics; these were selected to incorporate a variety of predicted interaction types and mechanisms of antibiotic activity. Three of the combinations we selected were predicted to synergize (greater antibiotic efficacy in combination than alone); three were predicted to antagonize (lower antibiotic efficacy in combination than alone), and four to interact additively or independently in Escherichia coli monoculture (16).

We tested the impact of antibiotic combinations in a previously described cross-feeding model system involving E. coli and Salmonella enterica. In this system, an E. coli methionine auxotroph produces acetate from lactose, and an S. enterica evolved mutant consumes the acetate while producing methionine (36–38). We can force the species to obligately cross-feed or grow independently by altering the growth medium. Previously, we used this system to demonstrate that cross-feeding can alter the ability of a resistant species to grow at high antibiotic concentrations; this effect is specific to metabolically interdependent communities and did not occur when we supplied the necessary nutrients for independent growth (36). We called this observation, that a cross-feeding pair can be inhibited by the most antibiotic susceptible species, the “weakest-link hypothesis” (36). Cross-feeding of both organic acids such as acetate and amino acids such as methionine are common in natural microbial food webs (39–41), making this a useful model system for testing general impacts of antibiotics on cross-feeding bacteria.

We used fractional inhibitory concentration indices (FICIs) to identify interactions between drug pairs. We first tested for antibiotic interactions in monocultures of E. coli and S. enterica. We then used our weakest-link hypothesis to predict the growth patterns of the coculture and the subsequent antibiotic interactions. It is worth mentioning that this study is specifically testing phenotypic rather than genetic antibiotic resistance (42–44). We have previously shown that growth in a cross-feeding community can change the ability of a given species to grow at high antibiotic concentrations (36). We therefore use phenotypic antibiotic resistance to refer to the phenotypic ability of a species to grow in a given concentration of antibiotic in a given growth/community condition.

In this study, we found that only three antibiotic combinations showed nonadditive interactions; however, our weakest-link hypothesis successfully predicted coculture growth and antibiotic interactions in these cases. Most importantly, we demonstrated that cross-feeding interactions can change antibiotic interactions from either monoculture. While more antibiotic combinations and a greater diversity of cross-feeding partnerships should be explored, these results suggest that the responses of individual community members to combination therapy might be sufficient to predict the antibiotic interactions in the larger microbial community.

## RESULTS

**FIG 1:**
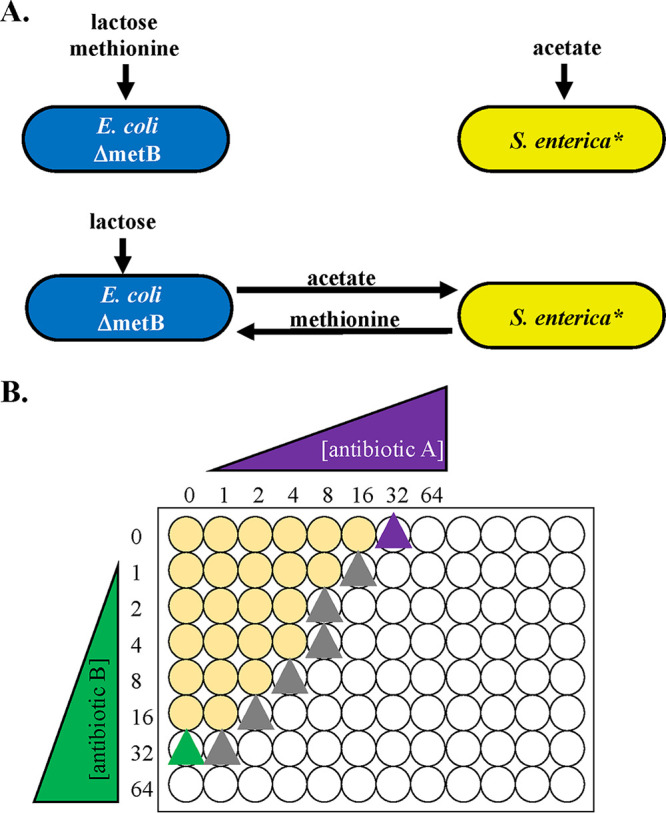
Antibiotic interaction experimental setup and hypotheses. (A) The two-species obligate cross-feeding system. When lactose is supplied, Escherichia coli uses it to produce acetate for Salmonella enterica, which produces methionine for E. coli. Each species can be grown in coculture or monoculture, depending on the metabolites supplied. (B) Setup for checkerboard assays. Seven antibiotic concentration wells plus one antibiotic-free well were developed for each antibiotic/species combination, with the MIC approximately in the middle of the gradient. Mid-log-phase cells were inoculated into plates containing species-specific growth medium and antibiotic at 2-fold dilutions. Cells were allowed to grow for 48 h at 30°C with shaking, and a Tecan plate reader was used to measure growth at an optical density of 600 nm (OD_600_). Growth was defined as an OD_600_ above 10% of the maximum OD_600_ obtained on each plate. Three replicates of each antibiotic/culture condition were obtained.

Based on previous results in E. coli (16), we tested 10 combinations of six antibiotics for synergy or antagonism in E. coli and S. enterica monocultures (Table 1). The mechanism of action for each of these antibiotics can be found in Table S1 in the supplemental material. We tested each combination in triplicate and calculated MICs, fractional inhibitory concentrations (FICs), and fractional inhibitory concentration indices (FICIs) using Loewe additivity (5) after 48 h of growth at 30°C (Fig. 1; see Fig. S1 in the supplemental material for additional detail on how the calculations were performed). To avoid over- or underinterpretation of the antibiotic interactions, we used the median FICI value for each plate and the mean value from each of the three replicate plates for each antibiotic combination.

**TABLE 1:** Antibiotic combinations used in the study and their predicted interactions in E. coli^*a*^

Interaction type	Antibiotic combinations
Synergy	Nalidixic acid and streptomycin, nalidixic acid and bleomycin, streptomycin and ciprofloxacin
Antagonism	Nalidixic acid and spectinomycin, nalidixic acid and doxycycline
Additive	Nalidixic acid and ciprofloxacin, ciprofloxacin and bleomycin, streptomycin and doxycycline, spectinomycin and doxycycline

a Based on Yeh et al. (16).

### Antibiotic interactions in monoculture differ from literature predictions.

**FIG 2:**
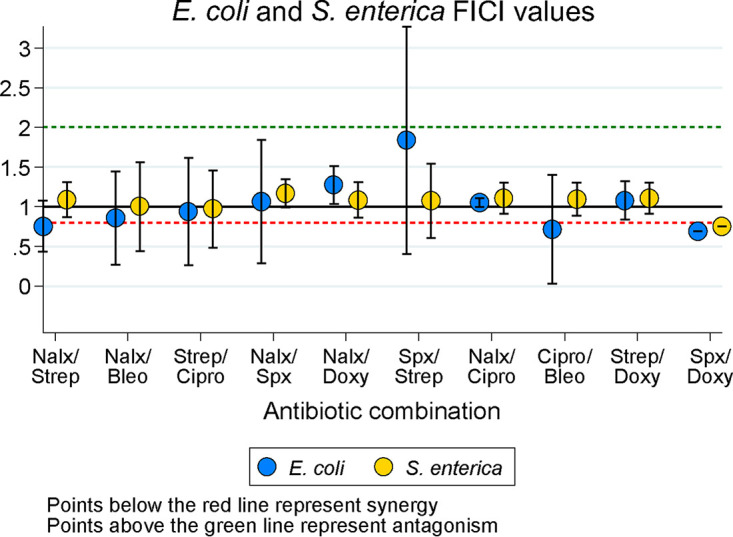
Fractional inhibitory concentration index (FICI) plots of E. coli and S. enterica monocultures across 10 antibiotic combinations. Each point represents the mean ± standard error (SE) of three replicate FICI values from three biological replicates. FICIs on each plate represent the median FICI value from the plate. Nalx, nalidixic acid; Strep, streptomycin; Bleo, bleomycin; Cipro, ciprofloxacin; Spx, spectinomycin; Doxy, doxycycline.

**FIG 3:**
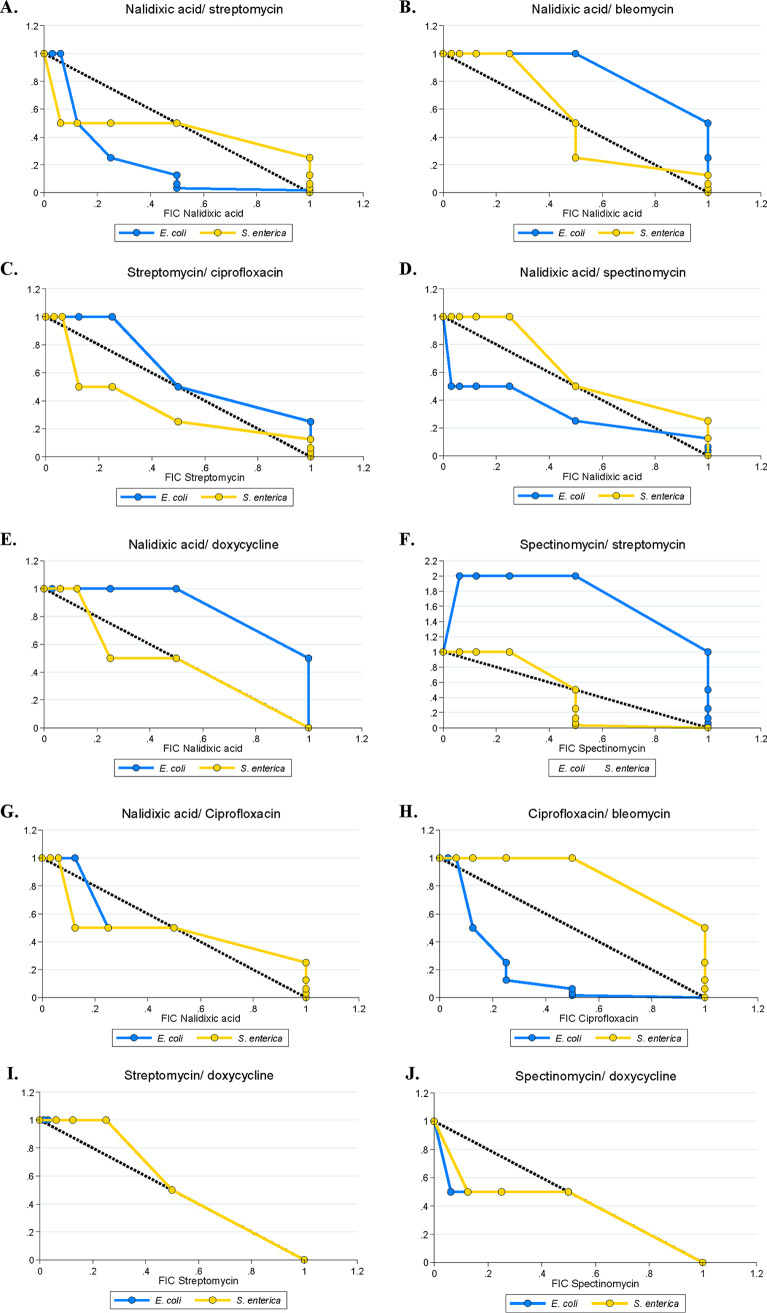
Representative isobolograms of E. coli and S. enterica monoculture fractional inhibitory concentrations (FICs) across 10 antibiotic combinations. FICs were calculated based on 48 h of 30°C growth, and growth was identified as any well which had an OD_600_ at least 10% of that of the highest-OD_600_ well on each plate. Each axis corresponds to a fractional inhibitory concentration (FIC) for the antibiotic pair. The black 1-1 line represent a perfectly independent interaction, a concave line toward the origin represents a synergistic interaction, and a convex line away from the origin represents an antagonistic interaction.

We first tested how the antibiotic combinations we selected would interact in our monocultures. We tested each antibiotic combination in triplicate for E. coli and S. enterica, then calculated the median FICI value for each plate and combination (Fig. 2). Our categories were designated as follows: an FICI value of <0.8 represents synergy, FICI values between 0.8 and 1 represent additive interactions, FICI values between 1 and 2 represent independent interactions, and an FICI value of ≥2 represents antagonism. These are less stringent than other FICI results because we chose median values to minimize the impact of plate-to-plate variation, and medians tend to bias FICI results away from detecting interactions. We also looked at isobolograms (Fig. 3) of each antibiotic combination for each species, to get a more visual/qualitative examination of interactions between antibiotics. Tables S2 and S3 in the supplemental material contain raw median and minimum FICI data, respectively.

The antibiotic interactions that we observed in monocultures were mostly additive, although we did see a few synergistic and antagonistic interactions. Our FICI (Fig. 2) and isobologram data (Fig. 3 and Fig. S2 in the supplemental material) showed additive/independent interactions for both species in nalidixic acid/bleomycin and streptomycin/ciprofloxacin. Nalidixic acid and streptomycin did synergize in E. coli, but not in S. enterica. Nalidixic acid/spectinomycin and nalidixic acid/doxycycline both showed additive interactions in both species; only spectinomycin/streptomycin showed antagonistic interactions. Finally, we observed that ciprofloxacin/bleomycin synergized in E. coli, and spectinomycin/doxycycline synergized in both species; however, this is more evident in the FICI data than in the isobolograms. The isobolograms suggest that low concentrations of doxycycline decrease the MIC of spectinomycin, but not vice versa; that is, doxycycline synergizes with spectinomycin to increase the latter’s potency, but spectinomycin does not change the effect of doxycycline. It is worth mentioning that these interactions differed from our predictions based on the literature (16); this likely illustrates the importance of strain-specific and assay-specific measurements of phenotypic resistance in bacteria.

### Antibiotic interactions in coculture match weakest-link predictions.

**FIG 4:**
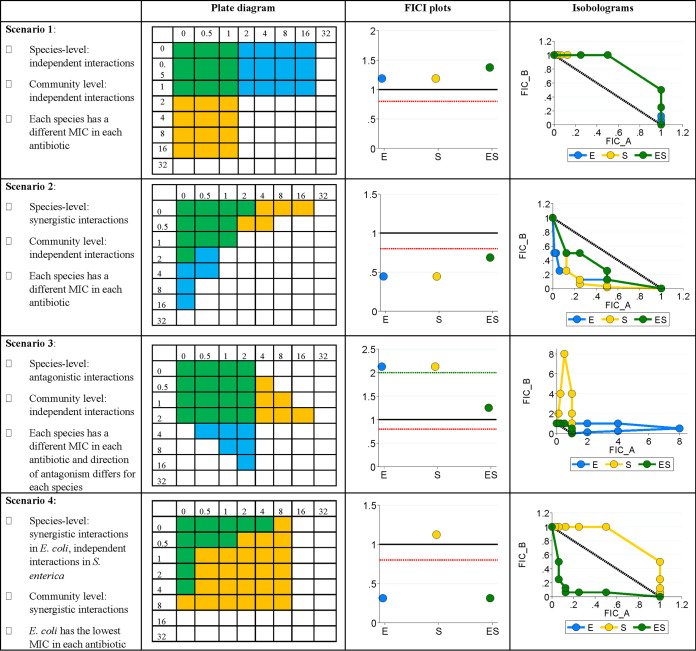
Antibiotic interactions at the species level versus the coculture level. In the plate diagrams (simulated data), blue cells represent concentrations under which only E. coli can grow; yellow cells represent concentrations under which only S. enterica can grow, and green cells represent concentrations under which the coculture can grow (i.e., concentrations under which both monocultures can grow). Antibiotic A is on the *y* axis, and antibiotic B is on the *x* axis. Points that fall below the red dotted line on FICI plots represent synergistic interactions; points that fall above the green dotted line represent antagonistic interactions. FICI plots and isobolograms were calculated based on the simulated data in plate diagrams (see Materials and Methods). Concave isoboles represent synergy, and convex isoboles represent antagonism.

**FIG 5:**
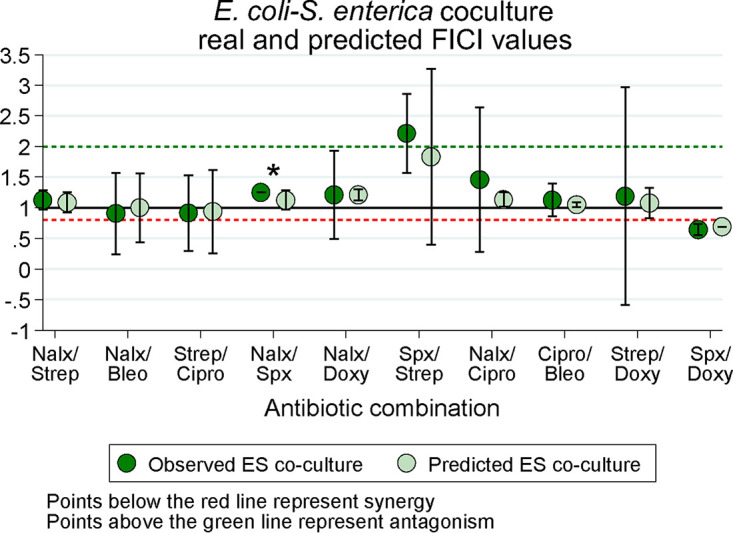
Fractional inhibitory concentration index (FICI) plots of predicted and actual cocultures across 10 antibiotic combinations. Each point represents the mean ± SE of three replicate FICI values from three biological replicates. FICIs on each plate represent the median FICI value from the plate. Asterisks represent a *P* value of <0.05 for predicted versus observed E. coli-S. enterica (ES) coculture; FICs were compared with a Mann-Whitney U test. *P* values can be found in Table S6 in the supplemental material. N, nalidixic acid; Strep, streptomycin; Bleo, bleomycin; Cipro, ciprofloxacin; Spx, spectinomycin; Doxy, doxycycline.

**FIG 6:**
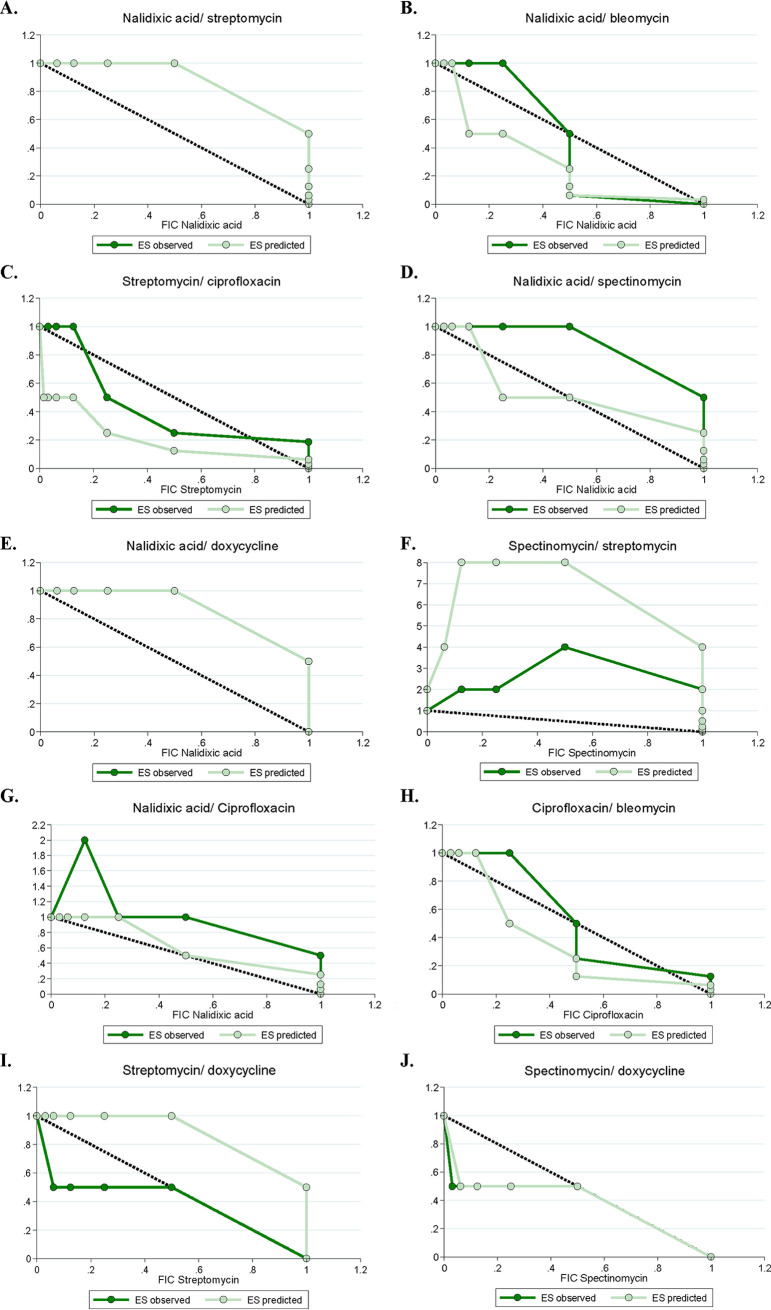
Representative isobolograms of predicted and observed coculture fractional inhibitory concentrations (FICs) across 10 antibiotic combinations. Predicted FICs were determined based on monoculture FICs and hypothesized weakest-link dynamics (i.e., coculture growth could only occur at concentrations of both antibiotics where both species could grow alone). Observed coculture FICs were calculated based on 48 h of 30°C growth, and growth was identified as any well which had an OD_600_ at least 10% that of the highest-OD_600_ well on each plate.

Previous work from our lab has shown that coculture growth in the presence of antibiotics is dependent on weakest-link dynamics (36). The weakest-link hypothesis predicts that the MIC of an obligately cross-feeding coculture is set by the MIC of the most antibiotic-susceptible species in the community. This hypothesis allows us to predict how antibiotics should interact in coculture based on how they interact in each monoculture (Fig. 4). To generate these predictions, we examined the monoculture growth patterns in each antibiotic combination (i.e., at which concentrations of each antibiotic monoculture growth occurred). We then generated a predicted growth pattern for the coculture in which growth would only occur at antibiotic concentrations where both species could grow. From this predicted growth pattern, we calculated FICIs and generated isobolograms; examples of these predictions these can be seen in Fig. 5 and 6, respectively.

According to our predictions, if one species is the most susceptible in both antibiotics, the coculture interaction typically matched that of the most susceptible monoculture. This is the case for nalidixic acid/bleomycin and nalidixic acid/ciprofloxacin (where S. enterica is the most susceptible), and for streptomycin/ciprofloxacin, spectinomycin/streptomycin, streptomycin/doxycycline, and spectinomycin/doxycycline (where E. coli is the most susceptible). Coculture predictions were somewhat more complicated for the other combinations (nalidixic acid/streptomycin, nalidixic acid/spectinomycin, nalidixic acid/doxycycline, and ciprofloxacin/bleomycin), where each species is the most susceptible to a different antibiotic. We were particularly interested in nalidixic acid/streptomycin, as these antibiotics synergize in E. coli (most susceptible to streptomycin) and interact independently in S. enterica (most susceptible to nalidixic acid). Based on the differences in MIC in these species in each antibiotic (see Table S4 in the supplemental material), we predicted an independent interaction in coculture. Similarly, in the ciprofloxacin/bleomycin combination, the antibiotics verged on antagonizing in E. coli and interacted independently in S. enterica; however, their MICs were similar in both antibiotics. This provided an opportunity to examine interactions in coculture where weakest-link dynamics might play less of a role.

After generating predicted FICIs based on our monoculture results and weakest-link dynamics, we tested antibiotic interactions in coculture. We then compared our predicted FICIs to those observed experimentally for each antibiotic combination. Qualitatively, our predictions based on the weakest-link hypothesis were accurate—the antibiotic interaction category (antagonism/synergy/additive) identified by predicted FICIs matched the interaction category identified by the observed FICIs (Fig. 5; see also Table S5 in the supplemental material for raw FICI data). This supports our hypothesis that weakest-link dynamics can be used to predict antibiotic interaction categories in coculture. The one exception to this was in the spectinomycin/streptomycin combination. While there was no statistical significance in this difference, (*P* = 0.37), we predicted an independent interaction and observed an antagonistic interaction. Interestingly, the isobologram suggested that antibiotics antagonized much more in coculture than we predicted. This suggests that weakest-link dynamics may not always predict coculture outcomes and that some other factor may be determining antibiotic interactions in this case. Quantitatively, our FICI predictions also matched that of our observed data (see Table S6 in the supplemental material for all *P* values), with one exception. The predicted FICI for the nalidixic acid/spectinomycin combination was significantly higher than predicted (*P* = 0.037), but this difference still resulted in independent interactions and so is likely not biologically significant. Overall, weakest-link dynamics were generally sufficient to both qualitatively and quantitatively predict antibiotic interactions in cocultures.

## DISCUSSION

The goal of this work was to test whether cross-feeding altered the impact of antibiotic combinations. We previously found that the antibiotic phenotypic resistance of a mutualistic coculture is set by the most antibiotic-susceptible species, a pattern we termed the weakest-link hypothesis. Here, we hypothesized that this effect could also change drug interaction patterns in antibiotic combinations. We tested previously identified antibiotic combinations in each of our monocultures and found that few of the predicted interactions held in our system. However, with the drug interactions we identified in monoculture, we used the weakest-link hypothesis to correctly predict the type of antibiotic interaction in the community context. The one exception to this was the spectinomycin/streptomycin combination, which antagonized more strongly in coculture than we predicted from monoculture. Our results provide a foundation for predicting the impact that ecological interactions have on pharmacological interactions.

We found that the antibiotic interactions that we observed in our monocultures did not match interactions that had previously been observed (16). In retrospect this is not surprising, as we used a different genotype of E. coli than was used by Yeh et al., and minimal rather than rich growth medium. Additionally, we used a yield-based checkerboard assay, while they used the growth rate-based dose-response curve measurement method (12). We elected to do a yield-based method because it allowed us to more highly parallelize our experiments. Parallelizing decreases plate-to-plate variation in cell density and growth phase, both of which are known to significantly impact phenotypic antibiotic resistance (45–47). Although we chose antibiotic combinations expected to evenly sample different types of interactions, under our conditions the antibiotics largely interacted additively. In future experiments, we will directly test how drug interactions in monocultures and cocultures change when using dose-response curves. This is particularly relevant for cocultures, as cross-feeding is known to alter growth rates of member species (48, 49). More broadly, the differences in observed and expected drug interactions highlight challenges the field faces with predicting drug interactions when they change with genotype, environment, and assay.

In spite of these challenges, we found that we were able to qualitatively predict the nature of drug interactions in a community context. Our predictions were informed by the weakest-link hypothesis, which posits that the most susceptible member of a cross-feeding system sets the antibiotic sensitivity for all members. We show that weakest-link dynamics should tend to ablate antagonistic/synergistic antibiotic interactions (Fig. 4). In line with this expectation, the antibiotic interaction for E. coli changed from synergistic in monoculture to additive in coculture for both nalidixic acid/streptomycin and ciprofloxacin/bleomycin. The union of antibiotic concentrations where the growth of both monocultures is permitted (i.e., the weakest-link predicted coculture growth pattern) will not always lead to additive drug interactions, however. We saw this in the difference between predicted and observed results for the interaction between spectinomycin and doxycycline in coculture. Most other drug combinations were additive in each monoculture and therefore remained additive in coculture. Further tests of the weakest-link hypothesis for drug combinations with nonadditive interactions are certainly warranted to further verify our results. However, our results provide broad support for the utility of the weakest-link hypothesis as at least a null model when predicting drug interactions in cross-feeding systems.

Our weakest-link predictions were not universally accurate in our experiments, however. Given that E. coli was the most susceptible to both streptomycin and spectinomycin, we predicted that the drug interaction in coculture would match the interaction in E. coli monoculture. Instead, the degree of antagonism increased in coculture. These particular antibiotics, which disrupt protein biosynthesis (50), may have sufficiently disrupted metabolism such that cross-feeding no longer occurred. That antibiotics can arrest growth rate (51, 52) and change the metabolic profile (53, 54) of cells is well known; therefore, further testing of antibiotics which target specific metabolic activity is needed. Additionally, while we were relatively successful in predicting coculture FICIs from monoculture data, we were much less successful in predicting qualitative antibiotic interactions from isobolograms. The isobologram of nalidixic acid/bleomycin in Fig. 6 provides a good example of this. The predicted coculture isobole showed additive-synergistic interactions; however, the observed coculture isobole showed synergistic interactions at low bleomycin FIC values. A similar pattern is seen with ciprofloxacin/bleomycin in the same figure. As polymicrobial infections are increasingly appreciated in clinical settings, it will be critical to develop new hypotheses and assays to further investigate antibiotic interactions in multispecies settings.

An outstanding challenge is how to translate our results to a clinical setting. As alluded to previously, the weakest-link hypothesis was developed in our E. coli*/*S. enterica model system but accurately predicts dynamics in a system relevant for disease in the lungs of cystic fibrosis patients (36). Drug-resistant Pseudomonas aeruginosa bacteria were inhibited by low antibiotic concentrations that directly inhibited anaerobes from which the pathogen cross-fed. This suggests that results from our system are likely to be generally predictive for cross-feeding bacteria; however, tests in additional cross-feeding systems are essential. Additionally, only a subset of infections are likely to involve cross-feeding. More broadly, our results argue for the importance of incorporating ecological interactions into treatment decisions. Weakest-link dynamics are just one of a growing list of ways that microbial interactions have been shown to alter the impact of antibiotics (55–57). Intriguing recent work suggests that drug effects in patients may be more accurately predicted by simply testing resistance profiles of mixed communities rather than monocultures (58).

Antibiotic cocktails have the potential to be powerful tools for precision medicine. However, for drug combinations to be employed effectively, we will need to overcome a range of challenges. For example, it will be critical to understand how differences in drug half-life and bioavailability impact effective dosages *in vivo*. Additionally, the dynamics of resistance evolution are likely to change in the face of multiple antibiotic pressure. Some research suggests that antibiotics which synergize in the short term may actually facilitate the evolution of resistance, while antagonistic interactions suppress resistance evolution (5, 59, 60). Our work suggests that ecological interactions can shape pharmacological interactions, so it will also be critical to better understand the variety of mechanisms through which this can happen and the variety of ways in which microbes interact in infection contexts. Despite these challenges, however, our work suggests that accurate prediction of drug interaction in cocultures is possible.

## MATERIALS AND METHODS

Our model microbial community has been previously described (37). Briefly, our system consists of an E. coli methionine auxotroph and an S. enterica strain which has been evolved to secrete excess methionine. In a lactose environment, E. coli metabolizes lactose to produce acetate for S. enterica, which in turn supplies methionine for E. coli. Each species can also be grown in monoculture by supplying E. coli with methionine and lactose and S. enterica with acetate.

We performed checkerboard assays (described below) with six antibiotics in 10 different combinations predicted to synergize (three combinations), antagonize (three combinations), or not interact (four combinations); see Table 1 for these combinations. For each drug combination, we tested E. coli and S. enterica in monocultures and the two-species obligate coculture. Each antibiotic combination/culture type was tested in triplicate. Seven 2-fold dilutions of each antibiotic, along with an antibiotic-free control for each, were used in orthogonal gradients on a 96-well plate such that the antibiotic concentrations increased from left to right and top to bottom. To avoid edge effects caused by evaporation, we did not use edge wells (these contained water and were not measured). Additionally, we placed permeable membranes on top of our plates to avoid evaporation and kept our incubator humidified. The first row and column of each plate were antibiotic-free wells for the vertically and horizontally distributed antibiotics, respectively. The MICs for each antibiotic were determined in the absence of the other antibiotic. Mid-log-phase cells (optical density [OD], ∼0.4) were grown up on the day of the experiment in species-specific Hypho growth medium (36) and 2 μl was inoculated into 194 μl fresh species-specific Hypho. For cooperative cocultures, 1 μl of each species was inoculated to establish a 1:1 species ratio. This allowed us to ensure that the same number of cells (∼5 × 10^5^ cells total per well) was inoculated in each well; this avoids density-dependent confounding effects on phenotypic antibiotic resistance. Antibiotic stocks were prepared within 2 days of the experiment such that 2 μl of stock could be added to each well to achieve the desired gradient concentrations. Plates were then incubated at 30°C with shaking for 48 h. A Tecan plate reader was then used to measure the OD at 600 nm (OD_600_) and species-specific fluorescence (cyan fluorescent protein [CFP] for E. coli and yellow fluorescent protein [YFP] for S. enterica). The MIC_90_ was then used to establish which wells showed growth. Any well that had an OD_600_ or fluorescent protein value above 10% of the highest plate value was considered growth. We used the highest plate value rather than the antibiotic-free well because we consistently saw a slight increase in OD_600_ in the cocultures at sublethal concentrations, possibly due to a low level of cell lysis and a subsequent boost for the cross-feeding partner (61, 62).

We used the Loewe additivity method to identify the nature of our antibiotic interactions as previously described (5). Briefly, we calculated the fractional inhibitory concentration (FIC) for antibiotics A and B as follows: FIC_A_ = (MIC_A in combination_/MIC_A alone_) and FIC_B_ = (MIC_B in combination_/MIC_B alone_). FIC values were obtained for each well at the edge of growth, as shown in Fig. 1 and Fig. S1 in the supplemental material. The FICI is the sum of FIC_A_ and FIC_B_ (63). As there are multiple FICI values per plate, we chose to report the median FICI value as the plate value. We did not use the minimum or maximum FICI value so that we would not overinterpret synergy or antagonism results, respectively (64). Minimum FICI values can be found in Table S3 in the supplemental material. Our cutoff values were designed as follows: an FICI value of <0.8 represents synergy, FICI values between 0.8 and 2 represent additive interactions, FICI values between 1 and 2 represent independent interactions, and an FICI value of ≥2 represents antagonism (63–66). Isobolograms were generated by plotting the FIC_A_ and FIC_B_ values as *x*, *y* coordinates. A straight line connecting the FIC values represents additive interactions, a concave line represents synergy, and a convex line represents antagonism.

Based on observed monoculture growth patterns (MICs and FICs in each antibiotic combination), we predicted coculture growth patterns assuming weakest-link dynamics; that is, cocultures should only grow at concentrations of both antibiotics where both species are able to grow in monoculture. These predictions are outlined in Fig. 4. In brief, the coculture is predicted to grow only where both species can grow individually (see plate diagrams). The impact of weakest-link dynamics on antibiotic interactions depends on whether the most susceptible species is the same or different in both antibiotics, and how the antibiotics interact with each species. In scenario 1, the most susceptible species differs in each antibiotic, but in both species the antibiotic effects are independent of each other; therefore, the antibiotics should also be independent in coculture. This is seen in the FICI plots (where the median FICI is around 1) and in the isobolograms (where the curve is around the 1-1 line). In scenario 2, the antibiotics synergize in both species, but because the most susceptible species differs in each antibiotic, the synergism is weakened (though still present) in coculture. In scenario 3, the antibiotics antagonize in both species. However, in E. coli, antibiotic B antagonizes antibiotic A (i.e., as the concentration of B increases, the MIC of A also increases), but not vice versa (i.e., the MIC of B does not change as the concentration of A increases). In S. enterica, antibiotic A antagonizes antibiotic B but not vice versa. This leads to a “cancelling out” of the antagonistic interactions in coculture and causes the antibiotics to interact independently. In scenario 4, E. coli is the most susceptible species in both antibiotics. Therefore, the coculture antibiotic interaction pattern exactly matches that of E. coli. We then calculated FICs and FICIs for these predicted coculture plates and compared them to our observed data. We then used a Mann-Whitney U test to compare predicted versus observed FICIs for our cocultures, as we had insufficient sample sizes to determine whether our data were normally distributed (a necessary assumption for performing a *t* test).

## Supplementary Material

Supplemental file 1
