# Supplementary material for: Weakest-Link Dynamics Predict Apparent Antibiotic Interactions in a Model Cross-Feeding Community
Source: Antimicrob Agents Chemother. 2020 Oct 20;64(11):e00465-20. doi: 10.1128/AAC.00465-20 (PMC7577160; doi:10.1128/AAC.00465-20)
Supplement: Supplemental file 1 [file AAC.00465-20-s0001.pdf]

Supplementary Information

Supplementary table S1. Mechanism of action of antibiotics used in this study.

Antibiotic	Mechanism
Bleomycin	Induces DNA breaks; may inhibit thymidine incorporation into DNA
Ciprofloxacin	Fluoroquinolone: binds DNA gyrase and topoisomerase IV
Nalidixic acid	Naphthyridone: binds DNA gyrase and topoisomerase IV
Doxycycline	Binds 30s ribosomal subunit to prevent protein biosynthesis
Spectinomycin	Binds 30s ribosomal subunit to prevent protein biosynthesis
Streptomycin	Binds 30s ribosomal subunit to prevent protein biosynthesis

A.

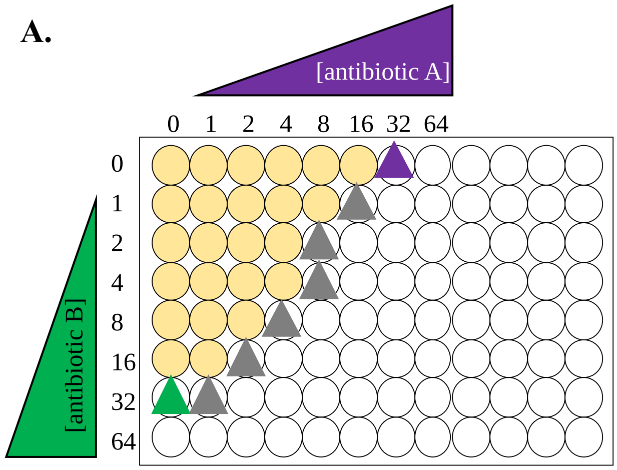

B.

$FIC_A = (MIC_{A\ with\ B}) / (MIC_{A\ alone})$
$FIC_B = (MIC_{B\ with\ A}) / (MIC_{B\ alone})$
$FICI = FIC_A + FIC_B$

**MIC_A**	**FIC_A**	**MIC_B**	**FIC_B**	**FICI**
0	0	32 ▲	1	1
1	0.03125	32	1	1.03125
2	0.0625	16	0.5	0.5625
4	0.125	8	0.25	0.375
8	0.25	4	0.125	0.375
8	0.25	2	0.0625	0.3125
16 ▲	0.5	1	0.03125	0.53125
32	1	0	0	1

Supplementary figure S1. Graphical representation of Loewe additivity calculation. **A.** Cartoon of bacterial growth after 48 hours across orthagonal antibiotic gradients in a 96 well plate. Yellow circles indicate growth; clear circles indicate no growth. The concentrations of antibiotics A and B are found along the top and left side of the plate, respectively. The purple triangle represents the MIC of antibiotic A in the absense of antibiotic B, and the green triangle represents the MIC of antibiotic B in the absense of antibiotic A. The grey triangles represent the concentrations of each antibiotic which are used to calculate the MIC of each antibiotic in the presence of the other. **B.** Table of values obtained from cartoon in panel **A** and used to calculate FIC and FICI values, as indicated in the box above the table.

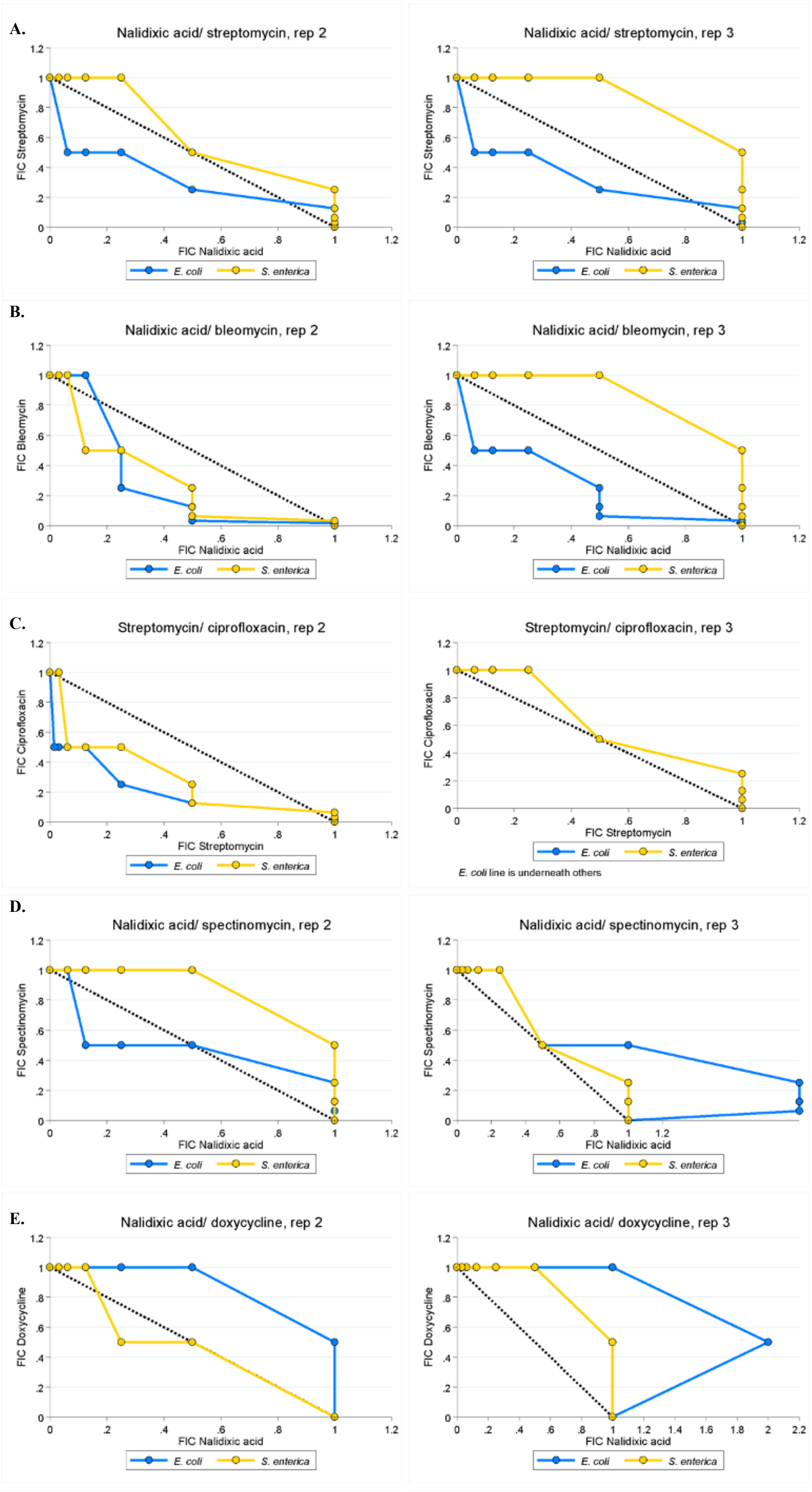
A.
Nalidixic acid/ streptomycin, rep 2
FIC Streptomycin
FIC Nalidixic acid
E. coli
S. enterica
Nalidixic acid/ streptomycin, rep 3
FIC Streptomycin
FIC Nalidixic acid
E. coli
S. enterica
B.
Nalidixic acid/ bleomycin, rep 2
FIC Bleomycin
FIC Nalidixic acid
E. coli
S. enterica
Nalidixic acid/ bleomycin, rep 3
FIC Bleomycin
FIC Nalidixic acid
E. coli
S. enterica
C.
Streptomycin/ ciprofloxacin, rep 2
FIC Ciprofloxacin
FIC Streptomycin
E. coli
S. enterica
Streptomycin/ ciprofloxacin, rep 3
FIC Ciprofloxacin
FIC Streptomycin
E. coli
S. enterica
E. coli line is underneath others
D.
Nalidixic acid/ spectinomycin, rep 2
FIC Spectinomycin
FIC Nalidixic acid
E. coli
S. enterica
Nalidixic acid/ spectinomycin, rep 3
FIC Spectinomycin
FIC Nalidixic acid
E. coli
S. enterica
E.
Nalidixic acid/ doxycycline, rep 2
FIC Doxycycline
FIC Nalidixic acid
E. coli
S. enterica
Nalidixic acid/ doxycycline, rep 3
FIC Doxycycline
FIC Nalidixic acid
E. coli
S. enterica

F.

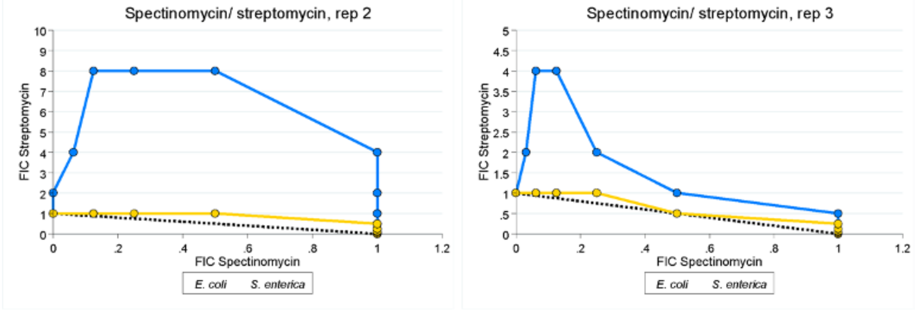

Spectinomycin/ streptomycin, rep 2
FIC Streptomycin
FIC Spectinomycin
E. coli
S. enterica
Spectinomycin/ streptomycin, rep 3
FIC Streptomycin
FIC Spectinomycin
E. coli
S. enterica

G.

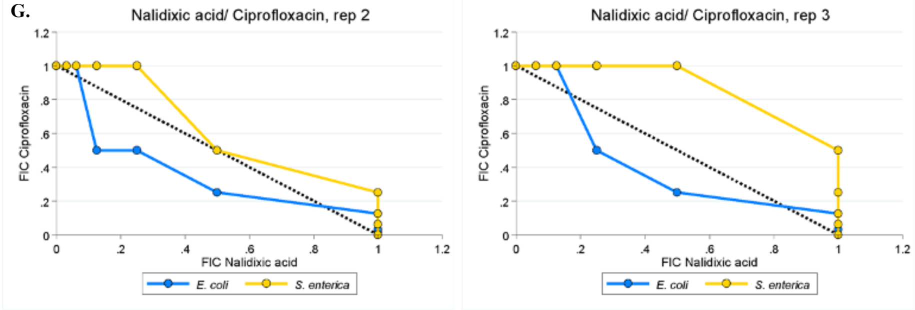

Nalidixic acid/ Ciprofloxacin, rep 2
FIC Ciprofloxacin
FIC Nalidixic acid
E. coli
S. enterica
Nalidixic acid/ Ciprofloxacin, rep 3
FIC Ciprofloxacin
FIC Nalidixic acid
E. coli
S. enterica

H.

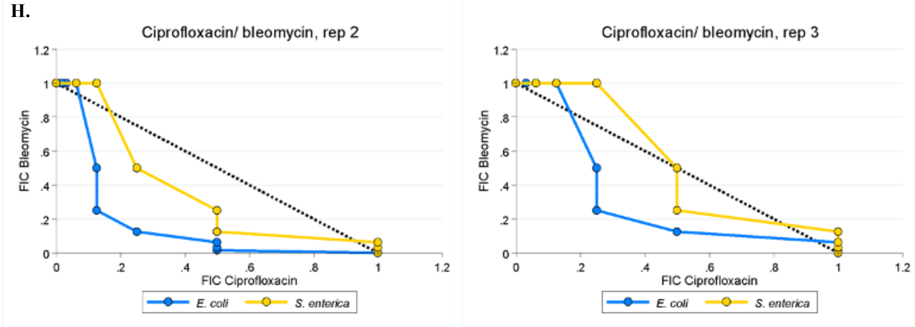

Ciprofloxacin/ bleomycin, rep 2
FIC Bleomycin
FIC Ciprofloxacin
E. coli
S. enterica
Ciprofloxacin/ bleomycin, rep 3
FIC Bleomycin
FIC Ciprofloxacin
E. coli
S. enterica

I.

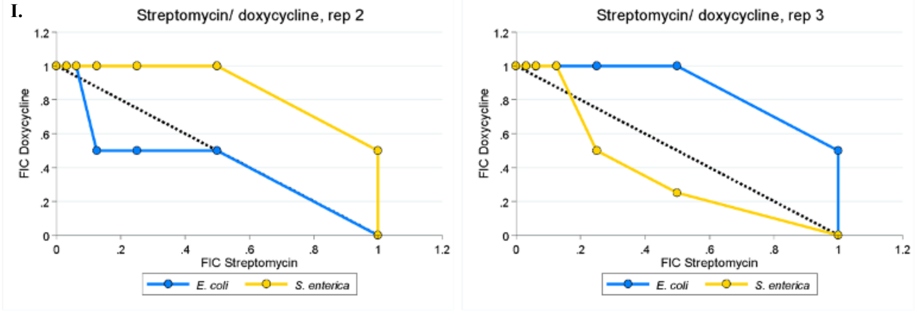

Streptomycin/ doxycycline, rep 2
FIC Doxycycline
FIC Streptomycin
E. coli
S. enterica
Streptomycin/ doxycycline, rep 3
FIC Doxycycline
FIC Streptomycin
E. coli
S. enterica

J.

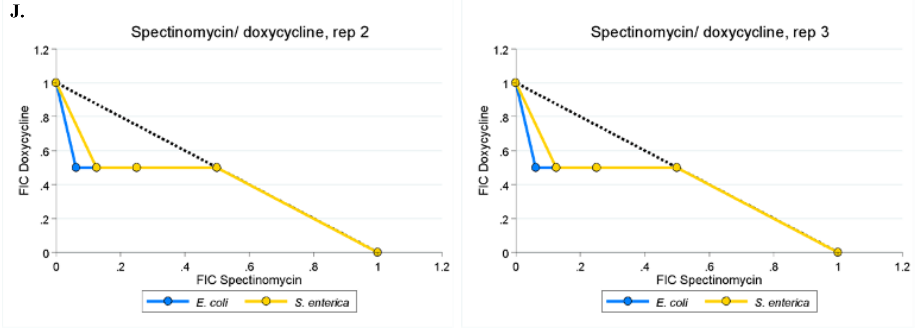

Spectinomycin/ doxycycline, rep 2
FIC Doxycycline
FIC Spectinomycin
E. coli
S. enterica
Spectinomycin/ doxycycline, rep 3
FIC Doxycycline
FIC Spectinomycin
E. coli
S. enterica

Supplementary figure S2. Isobolograms of replicates 2 and 3 for *E. coli* and *S. enterica* monoculture fractional inhibitory concentrations (FICs) across ten antibiotic combinations. Replicate 1 can be found in **Figure 3.**

Supplementary table S2. Median FICIs for *E. coli* and *S. enterica* in monoculture across ten antibiotic combinations and three replicates. FICIs for each replicate are the median FICI value per plate. FICI values below 0.8 are considered synergy; FICIs between 0.8 and 1 are additive interactions, FICIs between 1 and 2 are independent interactions, and FICIs above 2 are antagonistic interactions.

Species	Antibiotic combination	Rep 1	Rep 2	Rep 3
E. coli	Nalidixic acid/ streptomycin	0.75	0.625	0.8828125
	Nalidixic acid/ bleomycin	0.6875	0.75	1.125
	Streptomycin/ ciprofloxacin	1.125	0.625	1.0625
	Nalidixic acid/ spectinomycin	1.0625	0.75	1.375
	Nalidixic acid/ doxycycline	1.25	1.1875	1.375
	Spectinomycin/ streptomycin	2.5	1.5	1.5
	Nalidixic acid/ ciprofloxacin	1.0625	1.0234375	1.0625
	Ciprofloxacin/ bleomycin	0.546875	0.5625	1.03125
	Streptomycin/ doxycycline	1	1.046875	1.1875
	Spectinomycin/ doxycycline	0.6875	0.6875	0.6875
S. enterica	Nalidixic acid/ streptomycin	1.1875	1.015625	1.0625
	Nalidixic acid/ bleomycin	1.1875	0.75	1.0625
	Streptomycin/ ciprofloxacin	1.125	0.75	1.03125
	Nalidixic acid/ spectinomycin	1.25	1.125	1.125
	Nalidixic acid/ doxycycline	1.03125	1.03125	1.1875
	Spectinomycin/ streptomycin	1.25	0.875	1.09375
	Nalidixic acid/ ciprofloxacin	1.1875	1.09375	1.03125
	Ciprofloxacin/ bleomycin	1.1875	1.03125	1.0625
	Streptomycin/ doxycycline	1.1875	1.09375	1.03125
	Spectinomycin/ doxycycline	0.75	0.75	0.75

Supplementary table S3. Minimum FICIs for *E. coli* and *S. enterica* in monoculture across ten antibiotic combinations and three replicates. FICIs for each replicate are the minimum FICI value per plate. FICI values below 0.8 are considered synergy; FICIs between 0.5 and 1 are

additive interactions, FICIs between 1 and 2 are independent interactions, and FICIs above 2 are antagonistic interactions.

Species	Antibiotic combination	Rep 1	Rep 2	Rep 3
E. coli	Nalidixic acid/ streptomycin	0.5625	0.5	0.5625
	Nalidixic acid/ bleomycin	0.5625	0.5	1.015625
	Streptomycin/ ciprofloxacin	1	0.5	1
	Nalidixic acid/ spectinomycin	0.625	0.53125	1
	Nalidixic acid/ doxycycline	1.0625	1.03125	1.0625
	Spectinomycin/ streptomycin	1.0625	1.015625	1.015625
	Nalidixic acid/ ciprofloxacin	0.75	0.625	0.75
	Ciprofloxacin/ bleomycin	0.375	0.375	0.5
	Streptomycin/ doxycycline	0.625	1	1.03125
	Spectinomycin/ doxycycline	0.5625	0.5625	0.5625
S. enterica	Nalidixic acid/ streptomycin	1.0625	0.5625	1
	Nalidixic acid/ bleomycin	1.0625	0.5625	0.75
	Streptomycin/ ciprofloxacin	1	0.5625	0.625
	Nalidixic acid/ spectinomycin	1.0625	1	1
	Nalidixic acid/ doxycycline	0.75	0.75	1.03125
	Spectinomycin/ streptomycin	1	0.53125	1
	Nalidixic acid/ ciprofloxacin	1.0625	1	0.625
	Ciprofloxacin/ bleomycin	1.0625	0.625	0.75
	Streptomycin/ doxycycline	1.03125	1	0.75
	Spectinomycin/ doxycycline	0.625	0.625	0.625

E. coli	0	6.25	12.5	25	50	100	200	400
0								

A.

2								
4								
8								
16								
32								
64								
128								

B.

S. enterica	0	62.5	125	250	500	1000	2000	4000
0								
0.25								
0.5								
1								
2								
4								
8								
16								

C.

ES (predicted)	0	6.25	12.5	25	50	100	200	400
0								
0.25								
0.5								
1								
2								
4								
8								
16								

D.

ES (observed)	0	6.25	12.5	25	50	100	200	400
0								
0.25								
0.5								
1								
2								
4								
8								
16								

Supplementary figure S3. Example of developing predicted FICIs from replicate 1 of nalidixic acid/ spectinomycin combination. Growth patterns of *E. coli* (**A**) and *S. enterica* (**B**) monocultures were used to predict growth patterns for the co-culture (**C**). FICIs and isobolograms were developed from this predicted data as previously described, and these were compared to real data obtained from co-cultures (**D**).

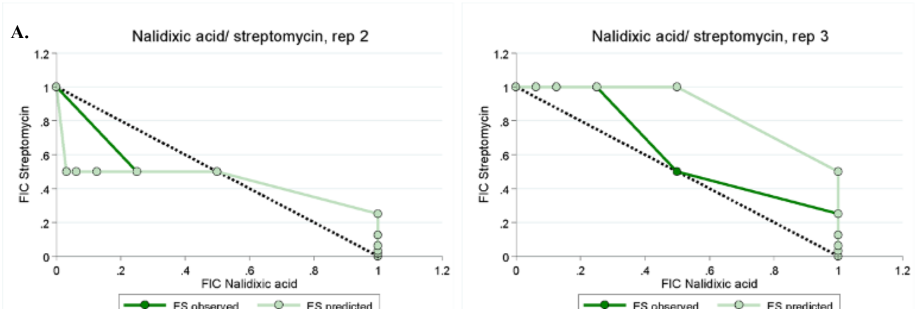

F.

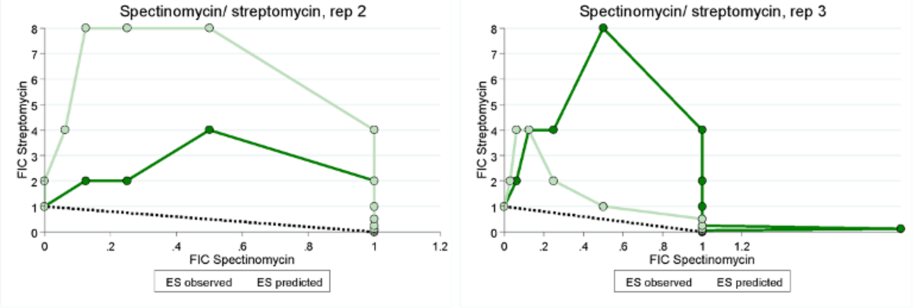
Spectinomycin/ streptomycin, rep 2
FIC Streptomycin
FIC Spectinomycin
ES observed
ES predicted
Spectinomycin/ streptomycin, rep 3
FIC Streptomycin
FIC Spectinomycin
ES observed
ES predicted

G.

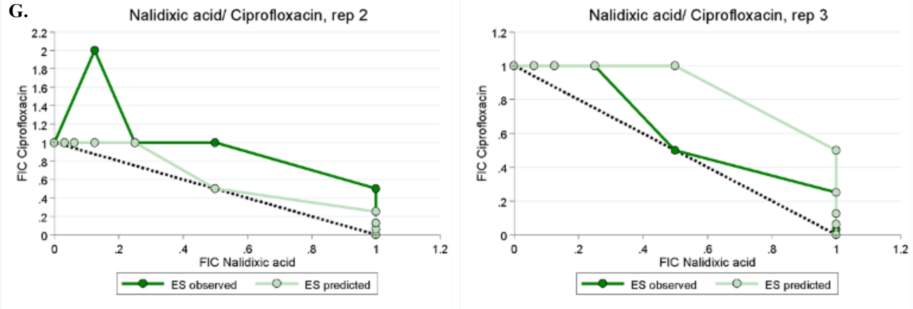
Nalidixic acid/ Ciprofloxacin, rep 2
FIC Ciprofloxacin
FIC Nalidixic acid
ES observed
ES predicted
Nalidixic acid/ Ciprofloxacin, rep 3
FIC Ciprofloxacin
FIC Nalidixic acid
ES observed
ES predicted

H.

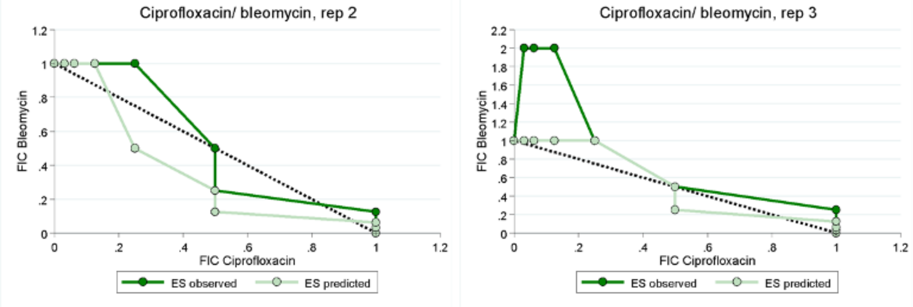
Ciprofloxacin/ bleomycin, rep 2
FIC Bleomycin
FIC Ciprofloxacin
ES observed
ES predicted
Ciprofloxacin/ bleomycin, rep 3
FIC Bleomycin
FIC Ciprofloxacin
ES observed
ES predicted

I.

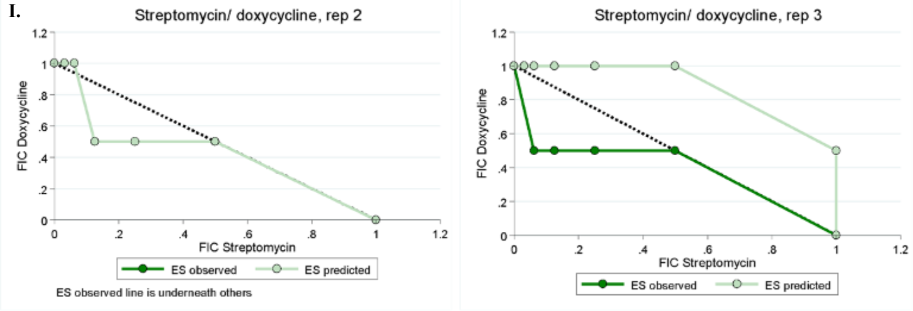
Streptomycin/ doxycycline, rep 2
FIC Doxycycline
FIC Streptomycin
ES observed
ES predicted
ES observed line is underneath others
Streptomycin/ doxycycline, rep 3
FIC Doxycycline
FIC Streptomycin
ES observed
ES predicted

J.

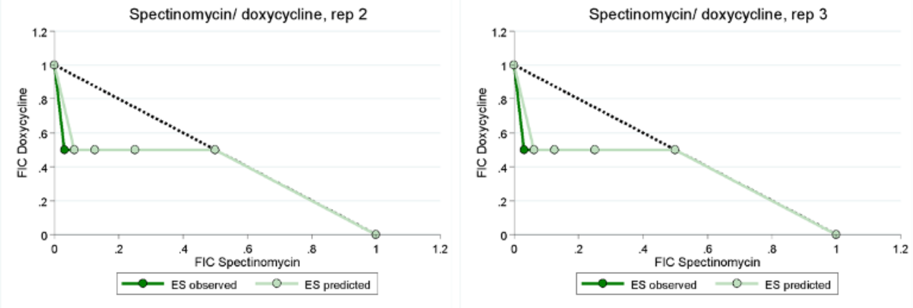
Spectinomycin/ doxycycline, rep 2
FIC Doxycycline
FIC Spectinomycin
ES observed
ES predicted
Spectinomycin/ doxycycline, rep 3
FIC Doxycycline
FIC Spectinomycin
ES observed
ES predicted

Supplementary figure S4. Isobolograms of replicates 2 and 3 for predicted and observed co-culture fractional inhibitory concentrations (FICs) across ten antibiotic combinations. Replicate 1 can be found in **Figure 6.**

Supplementary table S4. Minimum inhibitory concentrations (MICs) of each species in each antibiotic, predictions for co-cultures based on weakest link, and actual co-culture MICs. MICs were defined as the lowest concentration of antibiotic required to inhibit growth below 10% of the densest well (by OD600) within a plate. Medians and ranges are displayed. Predicted co-culture MICs are based on weakest link hypothesis (i.e. the co-culture will be limited by the least resistant monoculture).

**Antibiotic**	***E. coli* MIC**	***S. enterica* MIC**	**Predicted co-culture MIC**	**Observed co-culture MIC**
Bleomycin (µg/mL)	8 (2-8)	2 (1-2)	2 (1-2)	1 (0.5-2)
Ciprofloxacin (ng/mL)	16 (8-32)	16 (16-32)	16 (8-32)	16 (8-16)
Doxycycline (µg/mL)	0.25 (0.0625-0.25)	2.5 (2.5-5)	0.25 (0.0625-0.25)	0.25 (0.125-0.25)
Nalidixic acid (µg/mL)	32 (32-64)	8 (4-8)	8 (4-8)	2 (1-4)
Spectinomycin (µg/mL)	100 (100-200)	500 (500-1000)	100 (100-200)	100 (50-200)
Streptomycin (µg/mL)	1.5 (0.5-2)	160 (80-160)	1.5 (0.5-2)	0.5 (0.5-8)

Supplementary table S5. Observed fractional inhibitory concentration indices (FICIs) for each antibiotic combination in monoculture and co-culture, and predicted co-culture FICIs based on weakest link. FICIs are median values from three biological replicates each. Red cells represent synergistic interactions (median FICI<0.8); green cells represent antagonistic interactions (median FICI>2).

Antibiotic combination	*E. coli* FICI	*S. enterica* FICI	Predicted co-culture FICI	Observed co-culture FICI
Nalidixic acid/ streptomycin	0.75	1.06	1.13	1.13
Nalidixic acid/ bleomycin	0.75	1.06	1.06	0.88
Streptomycin/ ciprofloxacin	1.06	1.03	1.06	1.05
Nalidixic acid/ spectinomycin	1.06	1.13	1.13	1.25
Nalidixic acid/ doxycycline	1.25	1.03	1.19	1.38
Spectinomycin/ streptomycin	1.50	1.09	1.50	2.13
Nalidixic acid/ ciprofloxacin	1.06	1.09	1.13	1.25
Ciprofloxacin/ bleomycin	0.56	1.06	1.05	1.06
Streptomycin/ doxycycline	1.05	1.09	1.05	0.88
Spectinomycin/ doxycycline	0.69	0.75	0.69	0.63

Supplementary table S6. Mann-Whitney U statistical test results for predicted vs. observed FICI results.

Antibiotic combination	*P*-value for predicted vs. observed FICI
Nalidixic acid/ streptomycin	0.49
Nalidixic acid/ bleomycin	0.66
Streptomycin/ ciprofloxacin	0.50
Nalidixic acid/ spectinomycin	0.037
Nalidixic acid/ doxycycline	0.50
Spectinomycin/ streptomycin	0.37
Nalidixic acid/ ciprofloxacin	0.18
Ciprofloxacin/ bleomycin	0.10
Streptomycin/ doxycycline	0.51
Spectinomycin/ doxycycline	0.11
